# SGLT2 inhibitors in heart failure with reduced ejection fraction

**DOI:** 10.1186/s43044-021-00218-w

**Published:** 2021-10-24

**Authors:** Uday Sankar Das, Aritra Paul, Suvro Banerjee

**Affiliations:** 1 Apollo Gleneagles Hospitals, Kolkata, India; 2 Nil Ratan Sircar Medical College and Hospital, Kolkata, India; 3 Apollo Gleneagles Hospitals, 58, Canal Circular Road, Kolkata, West Bengal 700054 India

**Keywords:** Heart failure, Reduced ejection fraction, SGLT2 inhibitors, Diabetes, Renal disease

## Abstract

Sodium – glucose co-transporter 2 (SGLT2) inhibitors reduce blood glucose by inhibiting reabsorption of glucose from the proximal renal tubules. Initial studies showed that apart from reducing blood glucose they also reduce the combined endpoint of myocardial infarction, stroke, and cardiovascular death, hospitalization from heart failure, and occurrence of renal failure in patients with known cardiovascular disease or at high risk of developing cardiovascular disease. Recent studies have shown that these drugs also could be used in patients to treat heart failure or to slow the progression of renal failure, irrespective of whether the patients have diabetes or not. In this review, we discuss the clinical trial evidence for the use of SGLT2 inhibitors for the treatment of patients with heart failure with reduced ejection fraction and for the prevention of heart failure in patients with diabetes who are at high risk of cardiovascular events. We also discuss the plausible mechanisms of action for the cardiovascular beneficial effects of SGLT2 inhibitors. EMPA-REG OUTCOME TRIAL, DECLARE-TIMI 58, CANVAS, VERTIS-CV studies have shown that SGLT2 inhibitors namely empagliflozin, dapagliflozin, canagliflozin and ertugliflozin reduce the chances of hospitalisation in patients who have cardiovascular disease or at high risk of cardiovascular disease. The DAPA-HF study and the EMPEROR-REDUCED TRIAL have further shown that Dapagliflozin and Empagliflozin could be used to treat patients with heart failure, with or without diabetes. SGLT2 inhibitors provide us with a new armamentarium for treatment of patients with a triad of diabetes, heart or renal disease. Their mechanism of action in prevention or treatment of patients with heart failure however still remains speculative.

## Background

Heart failure (HF) is a clinical syndrome consisting of typical symptoms that may be accompanied by signs caused by a structural and/or functional cardiac abnormality that results in elevated intracardiac pressures and/or inadequate cardiac output at rest and/or during exercise. When this is accompanied by a reduced ejection fraction (EF) ≤ 40%, it is called heart failure with reduced ejection fraction (HFrEF).

Heart failure is one of the major causes of mortality in patients with type 2 diabetes mellitus (T2DM) [1, 2] and is highly prevalent in patients with diabetes [2, 3], occurring in more than one in five patients with diabetes aged over 65 years. Furthermore, T2DM is frequent in patients with HF, occurring in almost 40% of patients hospitalized for HF and up to 30% of those with chronic HF [4] in the community. Despite numerous available treatments for HF, the prognosis remains poor [5].

Concomitant T2DM confers a worse prognosis in HF as the risk of cardiovascular (CV) and all-cause mortality are significantly increased, independent of other risk factors [6, 7].

Although a modest cardiovascular benefit may be observed after a prolonged follow-up period [8], there was a concern that intensive glucose lowering or use of some of the glucose-lowering drugs may be associated with adverse cardiovascular outcomes [9]. Moreover, in a meta-analysis, no benefit on heart failure hospitalization or death was demonstrated with more intensive compared to less intensive glucose control [10]. Over the last decade, cardiovascular outcome trials have investigated several classes of new glucose-lowering agents.

Most of the dipeptidyl peptidase-4 inhibitors demonstrated cardiovascular safety in patients with T2DM. Interestingly and somewhat surprisingly, sodium–glucose co-transporter (SGLT2) inhibitors and glucagon-like peptide-1 receptor (GLP1) agonists were found to have cardiovascular benefits additionally.

## Main body

### SGLT2 inhibitors in the prevention of heart failure

Empagliflozin was the first SGLT2 inhibitor to show that it may prevent the development of heart failure in diabetic patients at risk of heart failure. In the EMPA-REG OUTCOME TRIAL [11], patients were randomized to receive empagliflozin 10 mg, empagliflozin 25 mg, or placebo in addition to the standard of care therapy for diabetes. In patients with T2DM and at high cardiovascular risk, empagliflozin reduced the risk of 3-point major adverse cardiovascular events (MACE) namely myocardial infarction, stroke, and cardiovascular death, all-cause death, and hospitalization for heart failure in comparison with the placebo. Consistent effects of empagliflozin were observed across all subgroups of patients.

The DECLARE-TIMI 58 trial [12] randomized patients with T2DM who were at risk for atherosclerotic cardiovascular disease to receive either dapagliflozin or placebo. The primary efficacy outcomes were MACE and a composite of cardiovascular death or hospitalization for heart failure. Dapagliflozin did not result in a significant difference in MACE than placebo but did result in a lower rate of cardiovascular death or hospitalization for heart failure.

Similarly, canagliflozin in the CANVAS trial [13] showed a lower risk of cardiovascular events than those who received the placebo, and Ertugliflozin in the VERTIS-CV trial [14] was non-inferior to placebo with respect to major adverse cardiovascular events. However, all the SGLT-2 inhibitors were associated with significant reduction in hospitalization for heart failure (Table 1).

**Table1:** Reduction in heart failure hospitalisation and CV death with empagliflozin, dapagliflozin,canagliflozin and ertugliflozin

	EMPA-REG OUTCOME (empagliflozin)	VERTIS CV (ertugliflozin)	CANVAS program (canagliflozin)	DECLARE TIMI (dapagliflozin)
	(RRR)	(RRR)	(RRR)	(RRR)
HHF	35% ↓	30% ↓	33% ↓	27% ↓
CV death	38% ↓	8% ↓	13% ↓	2% ↓

*HHF* hospitalisation for heart failure, *CV death* Cardiovascular death, *RRR* Relative risk reduction

In the CREDENCE study [15], patients with type 2 diabetes and albuminuric chronic kidney disease were randomised to receive canagliflozin or placebo. The primary outcome which was a composite of end-stage kidney disease, a doubling of the serum creatinine level, or death from renal or cardiovascular causes was significantly lower (a relative risk reduction of 30%) in the canagliflozin group than in the placebo group. The canagliflozin group also had a lower risk of cardiovascular death, myocardial infarction, or stroke.

Most patients in these trials did not have heart failure at baseline, so the benefit of treatment with an SGLT2 inhibitor largely reflected the prevention of incident heart failure. The reduction in the risk of hospitalization for heart failure was observed early after randomization which raised the possibility of mechanisms of action that differed from those usually postulated to explain the cardiovascular benefits of glucose-lowering therapies [16–20]. Besides, these agents slowed the progression of renal disease [11, 21, 22]. These effects on cardiovascular and renal outcomes are unlikely to be directly related to glycaemic control, suggesting that the benefits could also extend to patients without diabetes [11].

In these large scale randomized, placebo-controlled trials, the risk of hospitalization for heart failure was 30–35% lower among patients who received SGLT2 inhibitors than among those who received placebo [23], and this benefit was most striking in patients who had a left ventricular ejection fraction of 30% or less before treatment [24].

The 2019 European Society of Cardiology guidelines on diabetes, prediabetes, and cardiovascular diseases [25], the 2019 Heart Failure Association (HFA) position paper on the role and safety of new glucose-lowering medications [26], and the HFA clinical practice update on HF [27] recommend SGLT2 inhibitors to prevent HF hospitalization in patients with T2DM. The Heart Failure Association of European Society of Cardiology in October 2020 has also recommended empagliflozin, dapagliflozin, canagliflozin, and ertugliflozin for the prevention of heart failure hospitalization in patients with T2DM and established CV disease or at high CV risk.

### SGLT2 inhibitors in the treatment of heart failure

If SGLT2 inhibitors can prevent the development of heart failure in T2DM, can they be used for the treatment of heart failure in patients with T2DM? Moreover, will they also be useful for the treatment of patients without T2DM?

DAPA-HF study was the first to directly address these questions [8]. In this trial, 4744 Patients with heart failure NYHA class II to IV and ejection fraction of 40% or less were randomly assigned to receive either dapagliflozin 10 mg once daily or placebo in addition to recommended therapy. The risk of worsening heart failure (hospitalization or an urgent visit resulting in intravenous therapy for heart failure) or death from cardiovascular cause was lower among those who received dapagliflozin than among those who received placebo regardless of the presence or absence of diabetes. Among the secondary endpoints, composite of heart failure hospitalization or CV death, total number of heart failure hospitalization (including repeat admissions) and CV deaths, and all-cause deaths were also significantly reduced with dapagliflozin compared to placebo both in those with and without diabetes. The increase in total symptom score on the Kansas City Cardiomyopathy Questionnaire (indicating fewer symptoms) was also greater in the dapagliflozin group than in the placebo group between baseline and at 8th month.

Apart from the DAPA-HF trial, EMPEROR-REDUCED TRIAL is the only trial to date that included patients with symptomatic HFrEF, elevated natriuretic peptides, with or without T2DM. The trial was enriched for patients with more severe left ventricular function. The primary endpoint was CV death or heart failure hospitalization. Total (first and recurrent) heart failure hospitalization was a key secondary endpoint. There was a significant 25% reduction in the combined risk of cardiovascular death or first hospitalization for heart failure, and a significant 30% decrease in the total (first and recurrent) hospitalizations for heart failure. There was also significant improvement with empagliflozin in the exploratory endpoints such as urgent care visits for intravenous heart failure therapy and the Kansas City Cardiomyopathy score. Table 2 compares the clinical outcomes of DAPA-HF and EMPEROR-REDUCED trials.

**Table 2:** Comparison of the clinical outcomes of DAPA-HF and EMPEROR-REDUCED trials

Clinical outcomes	EMPEROR-Reduced (N = 3730)	DAPA-HF (N = 4744)
	Empagliflozin versus Placebo HR (CI)	Dapagliflozin versus Placebo HR (CI)
Cardiovascular death or HHF	0.75 (0.65–0.86)	0.75 (0.65–0.85)
Cardiovascular death	0.92 (0.75–1.12)	0.82 (0.69–0.98)
HHF	0.69 (0.59–0.81)	0.70 (0.59–0.83)

*HHF* hospitalisation for heart failure, *HR* Hazard ratio, *CI* confidence interval

### Biological mechanisms and effects of SGLT2 inhibitors in heart failure

**Fig. 1:**
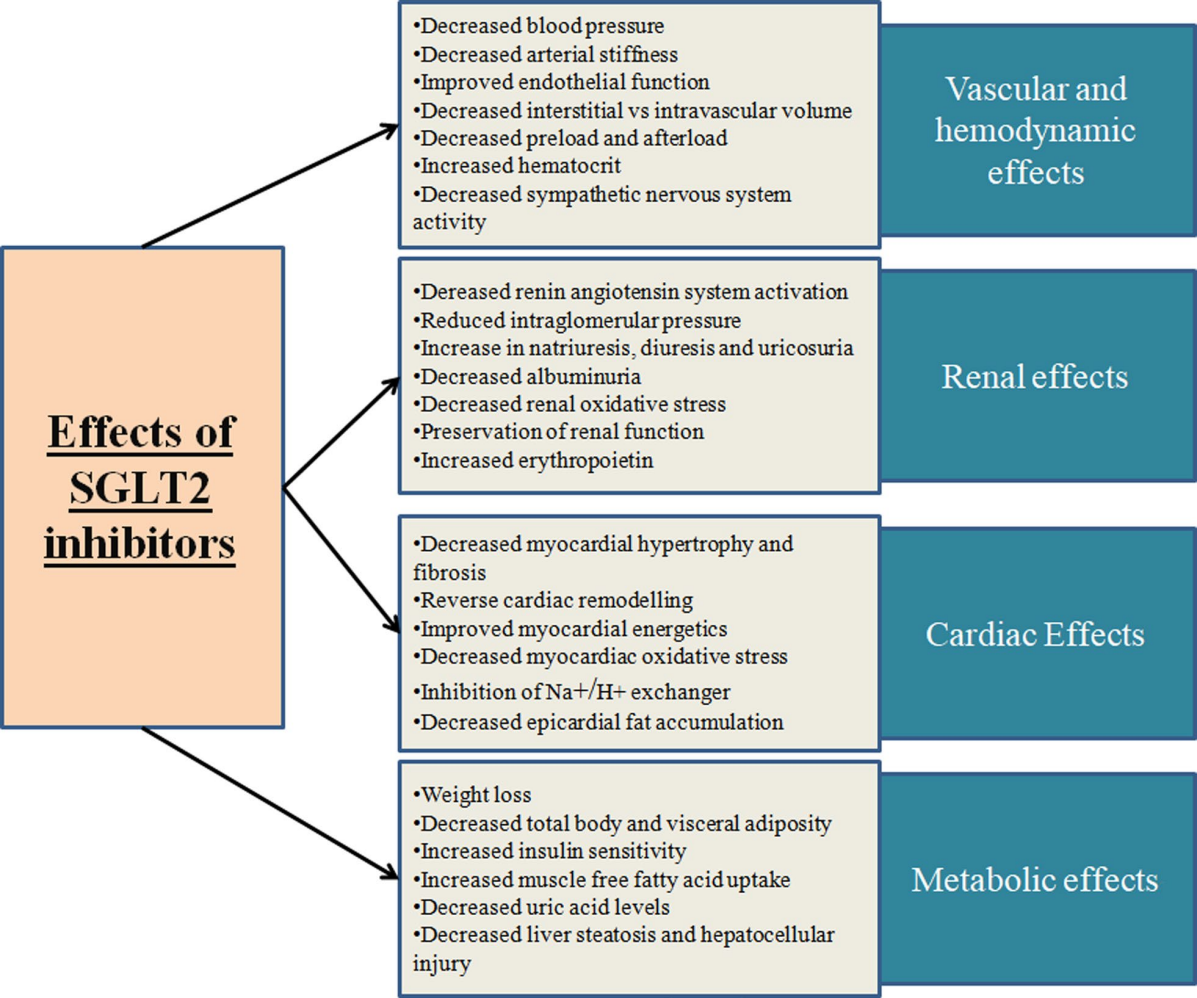
Figure summarizing the biological effects of SGLT2 inhibitors

The mechanisms of action of SGLT2 inhibitors in heart failure are still speculative although the drugs are shown to have several metabolic, hemodynamic, and organ-specific effects [Fig. 1]. In addition to glycosuria, SGLT2 inhibitors promote natriuresis and uricosuria [22, 28–32]. Other metabolic effects include increased insulin sensitivity and glucose uptake in muscle cells [32], decreased neoglucogenesis, and increased ketogenesis [12, 33]. These drugs also stimulate weight loss due to renal calorie loss in glycosuria [21, 29, 30] and a favorable impact on body fat distribution [33, 34].

A rise in hematocrit was also seen with SGLT2 inhibitor therapy. The hemodynamic effects are mediated by several mechanisms including osmotic diuresis, and plasma and interstitial fluid volume reduction, leading to a reduction in ventricular preload and afterload [28, 35, 36]. Furthermore, unlike diuretics, SGLT2 inhibitors seem to exert a greater reduction of interstitial fluid compared with plasma volume which may prevent plasma volume depletion and subsequent hypoperfusion occasionally observed with diuretics [37]. However these favorable metabolic and hemodynamic effects are unlikely to be solely responsible for the prevention and treatment of heart failure.

Another proposed mechanism for the beneficial effects of SGLT2 inhibitors is inhibition of the sodium-hydrogen exchanger (NHE1) activity which is up-regulated both in T2DM and heart failure [38]. By inhibiting the NHE1 receptors, SGLT2 inhibitors may protect the heart from toxic intracellular Ca2+ overload [39, 40].

SGLT2 inhibitors may also exert direct effects on myocardial metabolism [38, 41], and decrease myocardial oxidative stress [42]. Similar to T2DM, HF is characterized by a state of insulin resistance [43]. In the insulin-resistant heart, free fatty acids (FFA) are favored as an energy source over glucose which results in decreased cardiac metabolic efficiency (insufficient ATP production) [44]. By promoting a metabolic shift from FFA to glucose oxidation, SGLT2 inhibitors result in increased cardiac ATP production and prevent a decrease in cardiac function.

A benefit on ventricular remodeling was also demonstrated in patients with T2DM and coronary artery disease in the EMPA-HEART CardioLink-6 study, which showed a reduction in left ventricular (LV) mass index and improvement in diastolic function without changes in LV systolic function after 6 months of treatment with empagliflozin [16]. Furthermore, a significant reduction in LV mass in patients with T2DM was observed with dapagliflozin in the DAPA-HF trial, suggesting a possibility of reverse LV remodeling [21].

It is known that neurohormonal activation causes increased oxidative and other forms of cellular stress, which leads to dysfunction and loss of cardiomyocytes. Another postulated mechanism is that by inhibiting the energy surplus sensors SGLT2 inhibitors mimic cellular starvation and induce nutrient deprivation signals such as sirtuin 1 (SIRT1) which in turn inhibit activation of proinflammatory pathways, reduce cellular stress and promote autophagy [9]. This helps in reversing mitochondrial dysfunction and slowing cardiomyocyte dysfunction and cell loss. Other hypotheses include cardiac anti-fibrotic effects [28, 42], improved balance in adipokine secretion [45], beneficial effects on endothelial function [46], parameters of arterial stiffness and vascular resistance [47] as well as a reduction in sympathetic nervous system activity [48].

## Conclusions

SGLT2 inhibitors exemplify serendipity. Initially developed as glucose-lowering agents, they are found to have a more robust effect on heart failure and slowing of progression of renal dysfunction. Renal dysfunction often accompanies heart failure with reduced ejection fraction and these agents may be of particular value in such a clinical scenario. Trials are underway to answer whether SGLT2 inhibitors are also useful in patients with heart failure and preserved ejection fraction. One of them, the Emperor Preserved trial has already been published and the result shows that empagliflozin significantly reduces the combined primary endpoint of heart failure hospitalization and CV mortality in patients with heart failure and preserved ejection fraction regardless of whether T2DM was present or not [49].

Currently, SGLT2 inhibitors (empagliflozin, canagliflozin, dapagliflozin, ertugliflozin) are recommended to reduce the risk of HF hospitalization in T2DM patients with either established cardiovascular disease or at high cardiovascular risk Moreover, the recently published European Cardiology Society (ESC) 2021 guidelines have given a Class I recommendation for dapagliflozin and empagliflozin for the treatment of HFrEF, with or without T2DM [50]. Further large-scale clinical trials will provide the role of other SGLT2 inhibitors in the treatment of heart failure.

## Data Availability

All data generated or analysed during this study are included in this published article.

## Abbreviations

CV: Cardiovascular
FFA: Free fatty acids
GLP1: Glucagon-like peptide-1
HF: Heart failure
HFA: Heart Failure Association
HFrEF: Heart failure with reduced ejection fraction
LV: Left ventricle
MACE: Major adverse cardiovascular events
NHE1: Na^+^–H^+^ exchanger-1
NYHA: New York Heart Association
SGLT2: Sodium–glucose co-transporter 2
T2DM: Type II diabetes mellitus

## References

[1] Koudstaal S, Pujades-Rodriguez M, Denaxas S, Gho J, Shah AD, Yu N, Patel RS, Gale CP, Hoes AW, Cleland JG, Asselbergs FW, Hemingway H (2017). Prognostic burden of heart failure recorded in primary care, acute hospital admissions, or both: a population-based linked electronic health record cohort study in 2.1 million people. Eur J Heart Fail.

[2] Shah AD, Langenberg C, Rapsomaniki E, Denaxas S, Pujades-Rodriguez M, Gale CP, Deanfield J, Smeeth L, Timmis A, Hemingway H (2015). Type 2 diabetes and incidence of cardiovascular diseases: a cohort study in 1·9 million people. Lancet Diabetes Endocrinol.

[3] Bertoni AG, Hundley WG, Massing MW, Bonds DE, Burke GL, Goff DC (2004). Heart failure prevalence, incidence, and mortality in the elderly with diabetes. Diabetes Care.

[4] Seferović PM, Petrie MC, Filippatos GS, Anker SD, Rosano G, Bauersachs J, Paulus WJ, Komajda M, Cosentino F, de Boer RA, Farmakis D, Doehner W, Lambrinou E, Lopatin Y, Piepoli MF, Theodorakis MJ, Wiggers H, Lekakis J, Mebazaa A, Mamas MA (2018). Type 2 diabetes mellitus and heart failure: a position statement from the Heart Failure Association of the European Society of Cardiology. Eur J Heart Fail.

[5] Jones NR, Roalfe AK, Adoki I, Hobbs F, Taylor CJ (2019). Survival of patients with chronic heart failure in the community: a systematic review and meta-analysis. Eur J Heart Fail.

[6] Dauriz M, Targher G, Laroche C, Temporelli PL, Ferrari R, Anker S, Coats A, Filippatos G, Crespo-Leiro M, Mebazaa A, Piepoli MF, Maggioni AP, Tavazzi L, Heart Failure Long-Term Registry ESC-HFA (2017). Association between diabetes and 1-year adverse clinical outcomes in a multinational cohort of ambulatory patients with chronic heart failure: results from the ESC-HFA Heart Failure Long-Term Registry. Diabetes Care.

[7] Targher G, Dauriz M, Laroche C, Temporelli PL, Hassanein M, Seferovic PM, Drozdz J, Ferrari R, Anker S, Coats A, Filippatos G, Crespo-Leiro MG, Mebazaa A, Piepoli MF, Maggioni AP, Tavazzi L, ESC-HFA HF Long-Term Registry investigators, (2017). In-hospital and 1-year mortality associated with diabetes in patients with acute heart failure: results from the ESC-HFA Heart Failure Long-Term Registry. Eur J Heart Fail.

[8] Holman RR, Paul SK, Bethel MA, Matthews DR, Neil HA (2008). 10-year follow-up of intensive glucose control in type 2 diabetes. N Engl J Med.

[9] Udell JA, Cavender MA, Bhatt DL, Chatterjee S, Farkouh ME, Scirica BM (2015). Glucose-lowering drugs or strategies and cardiovascular outcomes in patients with or at risk for type 2 diabetes: a meta-analysis of randomised controlled trials. Lancet Diabetes Endocrinol.

[10] Turnbull FM, Abraira C, Anderson RJ, Byington RP, Chalmers JP, Duckworth WC, Evans GW, Gerstein HC, Holman RR, Moritz TE, Neal BC, Ninomiya T, Patel AA, Paul SK, Travert F, Woodward M, Control Group (2009). Intensive glucose control and macrovascular outcomes in type 2 diabetes. Diabetologia.

[11] Inzucchi SE, Zinman B, Fitchett D, Wanner C, Ferrannini E, Schumacher M, Schmoor C, Ohneberg K, Johansen OE, George JT, Hantel S, Bluhmki E, Lachin JM (2018). How does empagliflozin reduce cardiovascular mortality? Insights from a mediation analysis of the EMPA-REG OUTCOME Trial. Diabetes Care.

[12] Wiviott SD, Raz I, Bonaca MP, Mosenzon O, Kato ET, Cahn A, Silverman MG, Zelniker TA, Kuder JF, Murphy SA, Bhatt DL, Leiter LA, McGuire DK, Wilding J, Ruff CT, Gause-Nilsson I, Fredriksson M, Johansson PA, Langkilde AM, Sabatine MS (2019). Dapagliflozin and Cardiovascular Outcomes in Type 2 Diabetes. N Engl J Med.

[13] Neal B, Perkovic V, Mahaffey K, de Zeeuw D, Fulcher G, Erondu N (2017). Canagliflozin and cardiovascular and renal events in type 2 diabetes. N Engl J Med.

[14] Cannon CP, Pratley R, Dagogo-Jack S (2020). Cardiovascular outcomes with ertugliflozin in type 2 diabetes. N Engl J Med.

[15] Perkovic V, Jardine MJ, Neal B (2019). Canagliflozin and renal outcomes in type 2 diabetes and nephropathy. N Engl J Med.

[16] Verma S, Mazer CD, Yan AT, Mason T, Garg V, Teoh H, Zuo F, Quan A, Farkouh ME, Fitchett DH, Goodman SG, Goldenberg RM, Al-Omran M, Gilbert RE, Bhatt DL, Leiter LA, Jüni P, Zinman B, Connelly KA (2019). Effect of empagliflozin on left ventricular mass in patients with type 2 diabetes mellitus and coronary artery disease: the EMPA-HEART CardioLink-6 Randomized Clinical Trial. Circulation.

[17] Verma S, McMurray J (2018). SGLT2 inhibitors and mechanisms of cardiovascular benefit: a state-of-the-art review. Diabetologia.

[18] Inzucchi SE, Kosiborod M, Fitchett D, Wanner C, Hehnke U, Kaspers S, George JT, Zinman B (2018). Improvement in Cardiovascular Outcomes With Empagliflozin Is Independent of Glycemic Control. Circulation.

[19] Lytvyn Y, Bjornstad P, Udell JA, Lovshin JA, Cherney D (2017). Sodium glucose cotransporter-2 inhibition in heart failure: potential mechanisms, clinical applications, and summary of clinical trials. Circulation.

[20] Bonnet F, Scheen AJ (2018). Effects of SGLT2 inhibitors on systemic and tissue low-grade inflammation: the potential contribution to diabetes complications and cardiovascular disease. Diabetes Metab.

[21] McMurray J, Solomon SD, Inzucchi SE, Køber L, Kosiborod MN, Martinez FA, Ponikowski P, Sabatine MS, Anand IS, Bělohlávek J, Böhm M, Chiang CE, Chopra VK, de Boer RA, Desai AS, Diez M, Drozdz J, Dukát A, Ge J, Howlett JG (2019). Dapagliflozin in patients with heart failure and reduced ejection fraction. N Engl J Med.

[22] Neuen BL, Young T, Heerspink H, Neal B, Perkovic V, Billot L, Mahaffey KW, Charytan DM, Wheeler DC, Arnott C, Bompoint S, Levin A, Jardine MJ (2019). SGLT2 inhibitors for the prevention of kidney failure in patients with type 2 diabetes: a systematic review and meta-analysis. Lancet Diabetes Endocrinol.

[23] Mosenzon O, Wiviott SD, Cahn A, Rozenberg A, Yanuv I, Goodrich EL, Murphy SA, Heerspink H, Zelniker TA, Dwyer JP, Bhatt DL, Leiter LA, McGuire DK, Wilding J, Kato ET, Gause-Nilsson I, Fredriksson M, Johansson PA, Langkilde AM, Sabatine MS, Raz I (2019). Effects of dapagliflozin on development and progression of kidney disease in patients with type 2 diabetes: an analysis from the DECLARE-TIMI 58 randomised trial. Lancet Diabetes Endocrinol.

[24] Kato ET, Silverman MG, Mosenzon O, Zelniker TA, Cahn A, Furtado R, Kuder J, Murphy SA, Bhatt DL, Leiter LA, McGuire DK, Wilding J, Bonaca MP, Ruff CT, Desai AS, Goto S, Johansson PA, Gause-Nilsson I, Johanson P, Langkilde AM (2019). Effect of Dapagliflozin on Heart Failure and Mortality in Type 2 Diabetes Mellitus. Circulation.

[25] Cosentino F, Grant PJ, Aboyans V, Bailey CJ, Ceriello A, Delgado V, Federici M, Filippatos G, Grobbee DE, Hansen TB, Huikuri HV, Johansson I, Jüni P, Lettino M, Marx N, Mellbin LG, Östgren CJ, Rocca B, Roffi M, Sattar N (2020). 2019 ESC Guidelines on diabetes, pre-diabetes, and cardiovascular diseases developed in collaboration with the EASD. Eur Heart J.

[26] Seferović PM, Coats A, Ponikowski P, Filippatos G, Huelsmann M, Jhund PS, Polovina MM, Komajda M, Seferović J, Sari I, Cosentino F, Ambrosio G, Metra M, Piepoli M, Chioncel O, Lund LH, Thum T, De Boer RA, Mullens W, Lopatin Y (2020). European Society of Cardiology/Heart Failure Association position paper on the role and safety of new glucose-lowering drugs in patients with heart failure. Eur J Heart Fail.

[27] Seferovic PM, Ponikowski P, Anker SD, Bauersachs J, Chioncel O, Cleland J, de Boer RA, Drexel H, Ben Gal T, Hill L, Jaarsma T, Jankowska EA, Anker MS, Lainscak M, Lewis BS, McDonagh T, Metra M, Milicic D, Mullens W, Piepoli MF (2019). Clinical practice update on heart failure: pharmacotherapy, procedures, devices and patient management. An expert consensus meeting report of the Heart Failure Association of the European Society of Cardiology. Eur J Heart Fail.

[28] Lambers Heerspink HJ, de Zeeuw D, Wie L, Leslie B, List J (2013). Dapagliflozin a glucose-regulating drug with diuretic properties in subjects with type 2 diabetes. Diabetes Obes Metab.

[29] Neal B, Perkovic V, Mahaffey KW, de Zeeuw D, Fulcher G, Erondu N, Shaw W, Law G, Desai M, Matthews DR, CANVAS Program Collaborative Group (2017). Canagliflozin and cardiovascular and renal events in type 2 diabetes. N Engl J Med.

[30] Zhao Y, Xu L, Tian D, Xia P, Zheng H, Wang L, Chen L (2018). Effects of sodium-glucose co-transporter 2 (SGLT2) inhibitors on serum uric acid level: a meta-analysis of randomized controlled trials. Diabetes Obes Metab.

[31] Ferrannini E, Baldi S, Frascerra S, Astiarraga B, Heise T, Bizzotto R, Mari A, Pieber TR, Muscelli E (2016). Shift to fatty substrate utilization in response to sodium-glucose cotransporter 2 inhibition in subjects without diabetes and patients with type 2 diabetes. Diabetes.

[32] Ferrannini E, Solini A (2012). SGLT2 inhibition in diabetes mellitus: rationale and clinical prospects. Nat Rev Endocrinol.

[33] Waseda N, Satoh H, Yoshida C, Ikeda F, Kanazawa A, Watada H (2018) Effects of SGLT2 inhibitors on insulin secretion and insulin resistance–results from a cross-sectional study. Diabetes, 67(Suppl1),1187-P(abstr)

[34] Kuchay MS, Krishan S, Mishra SK, Farooqui KJ, Singh MK, Wasir JS, Bansal B, Kaur P, Jevalikar G, Gill HK, Choudhary NS, Mithal A (2018). Effect of empagliflozin on liver fat in patients with type 2 diabetes and nonalcoholic fatty liver disease: a randomized controlled trial (E-LIFT Trial). Diabetes Care.

[35] Cherney DZ, Perkins BA, Soleymanlou N, Maione M, Lai V, Lee A, Fagan NM, Woerle HJ, Johansen OE, Broedl UC, von Eynatten M (2014). Renal hemodynamic effect of sodium-glucose cotransporter 2 inhibition in patients with type 1 diabetes mellitus. Circulation.

[36] Yurista SR, Silljé H, Oberdorf-Maass SU, Schouten EM, Pavez Giani MG, Hillebrands JL, van Goor H, van Veldhuisen DJ, de Boer RA, Westenbrink BD (2019). Sodium-glucose co-transporter 2 inhibition with empagliflozin improves cardiac function in non-diabetic rats with left ventricular dysfunction after myocardial infarction. Eur J Heart Fail.

[37] Sha S, Polidori D, Heise T, Natarajan J, Farrell K, Wang SS, Sica D, Rothenberg P, Plum-Mörschel L (2014). Effect of the sodium glucose co-transporter 2 inhibitor canagliflozin on plasma volume in patients with type 2 diabetes mellitus. Diabetes Obes Metab.

[38] Yurista SR, Silljé H, van Goor H, Hillebrands JL, Heerspink H, de Menezes Montenegro L, Oberdorf-Maass SU, de Boer RA, Westenbrink BD (2020). Effects of sodium-glucose co-transporter 2 inhibition with empaglifozin on renal structure and function in non-diabetic rats with left ventricular dysfunction after myocardial infarction. Cardiovasc Drugs Ther.

[39] Uthman L, Baartscheer A, Bleijlevens B, Schumacher CA, Fiolet J, Koeman A, Jancev M, Hollmann MW, Weber NC, Coronel R, Zuurbier CJ (2018). Class effects of SGLT2 inhibitors in mouse cardiomyocytes and hearts: inhibition of Na^+^/H^+^ exchanger, lowering of cytosolic Na^+^ and vasodilation. Diabetologia.

[40] Iborra-Egea O, Santiago-Vacas E, Yurista SR, Lupón J, Packer M, Heymans S, Zannad F, Butler J, Pascual-Figal D, Lax A, Núñez J, de Boer RA, Bayés-Genís A (2019). Unraveling the molecular mechanism of action of empagliflozin in heart failure with reduced ejection fraction with or without diabetes. JACC Basic Transl Sci.

[41] Yamamoto C, Miyoshi H, Ono K, Sugawara H, Kameda R, Ichiyama M, Yamamoto K, Nomoto H, Nakamura A, Atsumi T (2016). Ipragliflozin effectively reduced visceral fat in Japanese patients with type 2 diabetes under adequate diet therapy. Endocr J.

[42] Brown A, Gandy S, McCrimmon R, Houston JG, Struthers AD, Lang CC (2020). A randomized controlled trial of dapagliflozin on left ventricular hypertrophy in people with type two diabetes: the DAPA-LVH trial. Eur Heart J.

[43] Li C, Zhang J, Xue M, Li X, Han F, Liu X, Xu L, Lu Y, Cheng Y, Li T, Yu X, Sun B, Chen L (2019). SGLT2 inhibition with empagliflozin attenuates myocardial oxidative stress and fibrosis in diabetic mice heart. Cardiovasc Diabetol.

[44] Paolisso G, De Riu S, Marrazzo G, Verza M, Varricchio M, D'Onofrio F (1991). Insulin resistance and hyperinsulinemia in patients with chronic congestive heart failure. Metabol Clin Exp.

[45] Pabel S, Wagner S, Bollenberg H, Bengel P, Kovács Á, Schach C, Tirilomis P, Mustroph J, Renner A, Gummert J, Fischer T, Van Linthout S, Tschöpe C, Streckfuss-Bömeke K, Hasenfuss G, Maier LS, Hamdani N, Sossalla S (2018). Empagliflozin directly improves diastolic function in human heart failure. Eur J Heart Fail.

[46] Packer M (2018). Do sodium-glucose co-transporter-2 inhibitors prevent heart failure with a preserved ejection fraction by counterbalancing the effects of leptin? A novel hypothesis. Diabetes Obes Metab.

[47] Shigiyama F, Kumashiro N, Miyagi M, Ikehara K, Kanda E, Uchino H, Hirose T (2017). Effectiveness of dapagliflozin on vascular endothelial function and glycemic control in patients with early-stage type 2 diabetes mellitus: DEFENCE study. Cardiovasc Diabetol.

[48] Chilton R, Tikkanen I, Cannon CP, Crowe S, Woerle HJ, Broedl UC, Johansen OE (2015). Effects of empagliflozin on blood pressure and markers of arterial stiffness and vascular resistance in patients with type 2 diabetes. Diabetes Obes Metab.

[49] Anker SD, Butler J, Filippatos G, Ferreira JP, Bocchi E, Böhm M, Brunner-La Rocca HP, Choi DJ, Chopra V, Chuquiure-Valenzuela E, Giannetti N, Gomez-Mesa JE, Janssens S, Januzzi JL, Gonzalez-Juanatey JR, Merkely B, Nicholls SJ, Perrone SV, Piña IL, Ponikowski P (2021). Empagliflozin in heart failure with a preserved ejection fraction. N Engl J Med.

[50] McDonagh T, Metra M, Adamo M, Gardner R, Baumbach A, Böhm M (2021). 2021 ESC Guidelines for the diagnosis and treatment of acute and chronic heart failure. Eur Heart J.

